# Juvenile hormone pathway in honey bee larvae: A source of possible signal molecules for the reproductive behavior of *Varroa destructor*

**DOI:** 10.1002/ece3.7125

**Published:** 2020-12-21

**Authors:** Cristian M. Aurori, Alexandru‐Ioan Giurgiu, Benjamin H. Conlon, Chedly Kastally, Daniel S. Dezmirean, Jarkko Routtu, Adriana Aurori

**Affiliations:** ^1^ Faculty of Animal Science and Biotechnology University of Agriculture Sciences and Veterinary Medicine Cluj‐Napoca Romania; ^2^ Molecular Ecology Institute of Biology/Zoology Martin‐Luther‐University Halle‐Wittenberg Halle Germany; ^3^ Section for Ecology and Evolution Department of Biology University of Copenhagen Copenhagen Denmark; ^4^ Department of Ecology and Genetics and Biocenter Oulu University of Oulu Oulu Finland; ^5^ Advanced Horticultural Research Institute of Transylvania University of Agriculture Sciences and Veterinary Medicine Cluj‐Napoca Romania

**Keywords:** drone, ecdysteroid, jhamt, kairomone, larvae, methyl farnesoate

## Abstract

The parasitic mite *Varroa destructor* devastates honey bee (*Apis mellifera*) colonies around the world. Entering a brood cell shortly before capping, the *Varroa* mother feeds on the honey bee larvae. The hormones 20‐hydroxyecdysone (20E) and juvenile hormone (JH), acquired from the host, have been considered to play a key role in initiating *Varroa's* reproductive cycle. This study focuses on differential expression of the genes involved in the biosynthesis of JH and ecdysone at six time points during the first 30 hr after cell capping in both drone and worker larvae of *A. mellifera*. This time frame, covering the conclusion of the honey bee brood cell invasion and the start of *Varroa's* ovogenesis, is critical to the successful initiation of a reproductive cycle. Our findings support a later activation of the ecdysteroid cascade in honey bee drones compared to worker larvae, which could account for the increased egg production of *Varroa* in *A. mellifera* drone cells. The JH pathway was generally downregulated confirming its activity is antagonistic to the ecdysteroid pathway during the larva development. Nevertheless, the genes involved in JH synthesis revealed an increased expression in drones. The upregulation of *jhamt* gene involved in methyl farnesoate (MF) synthesis came into attention since the MF is not only a precursor of JH but it is also an insect pheromone in its own right as well as JH‐like hormone in Acari. This could indicate a possible kairomone effect of MF for attracting the mites into the drone brood cells, along with its potential involvement in ovogenesis after the cell capping, stimulating *Varroa's* initiation of egg laying.

## Graphical abstract

In an effort to answer a long‐standing question on how hormonal interactions in the honey bee larvae can sustain the parasite *Varroa destructor* reproduction, the current study focused on differential expression analysis of the genes involved in the Juvenile hormone (JH) and ecdysteroid associated pathways. All the results were discussed considering the *V. destructor* related reproductive behavior. The results revealed the higher expression in drones relative to workers for the jhamt gene involved in methyl farnesoate (MF) synthesis. MF is the precursor of JH, also an insect pheromone and the JH‐like hormone in Acari. These results suggest a possible kairomone effect of MF for attracting the mites into the drone brood cells, along with its potential involvement in ovogenesis after the cell sealing, stimulating Varroa's fertility.

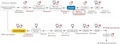

## 1 INTRODUCTION

*Varroa destructor* represents the most serious threat to the western honey bee *Apis mellifera* colonies in almost every biogeographic region on earth (Dietemann et al., 2013; Neumann & Carreck, 2010; Steinhauer et al., 2018). In *A. cerana*, *Varroa's* original host, the mite's development is restricted to the drone cell, a result of both behavioral and physiological traits (Martin, 1994; Rosenkranz et al., 1993 **).** In *A. mellifera*, *Varroa* also shows a preference for the drone cells where increased fecundity results in higher fitness for the infesting mother (Fuchs, 1992). *Varroa's* reproductive cycle begins when a mother mite enters into a brood cell shortly before capping. Roughly 6 hr postcapping, the mother mite's oocytes start their maturation (Garrido et al., 2000). Vitellogenesis, however, only occurs after the *Varroa* mother begins feeding on the pupa (Steiner et al., 1994, 1995). These fundamental steps in *Varroa's* reproduction take place during the LS1 stage of honey bee larvae (Cabrera Cordon et al., 2013), which experiences sudden changing titers of Juvenile hormone (JH) and ecdysteroids (Hartfelder & Engels, 1998). It is only through a tight synchronization with the host honey bee's development that *Varroa* is able to ensure a successful reproductive cycle (Frey et al., 2013; Garrido et al., 2000). Distinct mechanisms of *Varroa* resistance resulting from either a delay in the initiation of egg laying or in reduced fertility for the mite (Locke et al., 2012) raise a question—which of the host‐derived factors at this particular time frame could be responsible for these effects?

In holometabolous insects, the transition from larvae to pupae is a well‐orchestrated process controlled by the interplay between JH and ecdysteroids (Riddiford, 2012). JH is required for normal larval development while suppressing the initiation of metamorphosis when increased ecdysteroid production triggers molting. The presence of JH ensures the “status quo” in larvae, allowing the ecdysteroids to initiate molting but prevents its action for inducing metamorphosis (Riddiford, 1996). In *Manduca sexta*, a well‐established experimental model for the study of JH and ecdysteroids functions, the JH titer begins to decrease to an undetectable level during the last larval instar. This decrease in JH allows the release of ecdysteroids from the prothoracic gland. In the absence of JH, ecdysteroids cause the cessation of larval feeding and induce the prepupal commitment processes (Riddiford, 2012). In honey bees, a similar decrease in JH titer in the hemolymph has been encountered right before the transition from the feeding (LF3) to the spinning stage (LS1) of the 5th instar larvae (Rachinsky et al., 1990). In vivo studies showed (Feldlaufer et al., 1985; Rachinsky et al., 1990) and subsequent in vitro tests confirmed (Hartfelder, 1993) that during this particular developmental stage the ecdysteroids are present, although at rather low level. The release timing of ecdysteroids from the prothoracic gland is similar for both workers and queens—at the beginning of the LS1 – with a higher level in the latter. The ecdysteroids’ titer in drone brood during the spinning phase (LS1–LS3) has been found to be at an intermediate level between the female castes (Hartfelder & Engels, 1998).

The ecdysteroid synthesis pathway requires the engagement of a chain of enzymes coded by the Halloween genes: *neverland*, nonmolting glossy/*shroud* (Yamazaki et al., 2011), *spookiest*, *phantom*, *disembodied*, *shadow,* and *shade* (Gilbert, 2004; KEGG—Kanehisa et al., 2016) (Table 1). In honey bees, detailed expression of Halloween genes in pupae is reported by only a few studies (Conlon et al., 2019; Yamazaki et al., 2011). However, despite *Varroa* lacking a complete set of Halloween genes (Techer et al., 2019), nobody has systematically investigated the link between JH and ecdysteroid gene expression in honey bee prepupae and a *Varroa's* reproductive cycle.

**TABLE 1:** Molting hormone pathway genes and corresponding enzymes as described in KEGG, NCBI, and UniProt databases

Gene symbol	Gene description (NCBI)	Also known as (KEGG or NCBI)	NCBI Accession number (gene ID)	Enzyme NCBI‐Protein ID	Function (KEGG, UniProt)
Molting hormone pathway					
LOC107965828	Cholesterol 7‐desaturase	neverland	107965828	XP_026296485	Cholesterol 7‐desaturase
LOC724348	Short‐chain dehydrogenase/reductase	shroud	724348		
LOC410495	Cytochrome P450 307a1	CYP307B1; spookiest	410495	XP_026300894	
LOC408398	Cytochrome P450 306a1	CYP306A1; Phm; phantom	408398	XP_006557887	
LOC727118	Cytochrome P450 302a1, mitochondiral	CYP302A1; disembodied; Dib	727118	XP_026300415	
LOC411893	Cytochrome P450 315a1, mitochondiral	CYP315A1; shadow; Sad	411893	XP_006563995	
Cyp314a1	Cytochrome P450 314A1	shade; GB13998	411057	NP_001035347	Oxydoreductase
LOC410405	Cytochrome P450 18a1	cyp18a1	410405	XP_393885	Oxydoreductase

The pathway of JH synthesis involves the activity of 5 genes (KEGG data—Kanehisa et al., 2016) (Table 2). The first one, *4‐nitrophenylphosphatase* (FPPP), catalyzes the hydrolysis of farnesyl pyrophosphate to farnesol (Cao et al., 2009), which is then converted by *farnesol dehydrogenase* and *retinal dehydrogenase 1* to farnesal and farnesoic acid, respectively (Nyati et al., 2013). *JH acid O‐methyltransferase* (*jhamt*) transfers a methyl group from S‐adenosyl‐L‐methionine to the carboxyl group of farnesoic acid acids to produce methyl farnesoate (MF) (Niwa et al., 2008), which is converted by *MF epoxidase* (MFE) to JH (Bomtorin et al., 2014). Two enzymes, *JH epoxide hydrolase* and *JH esterase*, are responsible for JH degradation in honey bees, the latter being considered as having the primary role (Mackert et al., 2008, 2010).

**TABLE 2:** Juvenile hormone pathway genes and corresponding enzymes as described in KEGG, NCBI, and Uniprot databases

Gene symbol	Gene description (NCBI)	Also known as (KEGG or NCBI)	NCBI Accession number (gene ID)	Enzyme NCBI‐Protein ID	Mol Function (Kegg, UniProt)
Juvenile hormone pathway					
LOC551405	4‐nitrophenylphosphatase	FPPP	551405	XP_623802	Phosphoric‐monoester hydrolase
LOC725489	Farnesol dehydrogenase		725489	XP_016773395	Farnesol dehydrogenase (NADP+)
LOC408559	Retinal dehydrogenase 1		408559	XP_392104	Aldehyde dehydrogenase (NAD+)
LOC724216	Juvenile hormone acid O‐methyltransferase	jhamt	724216	NP_001314896	Methyltransferase
LOC551179	Methyl farnesoate epoxidase	CYP15A1	551179	NP_001314895	Methyl farnesoate epoxidase/farnesoate epoxydase
Jhe	Juvenile hormone esterase	Jhe; Est; Dpa1; GB15327	406066	NP_001011563	Hydrolase
LOC406152	Juvenile hormone epoxide hydrolase 1	JHEH	406152	XP_394822	Insect hormone biosynthesis

*Varroa's* reproductive success seems to be affected by several co‐occurring biochemical factors in the host, of which JH has been thought to play an important role. Earlier studies have shown its general role in increasing mite fertility, with a bias toward drones as a source (Hanel & Koeniger, 1986), but later findings questioned its role (Cabrera Cordon et al., 2013; Rosenkranz et al., 1990, 1993). Recent findings now suggest the ecdysteroids play an important role in the life cycle of *Varroa* (Cabrera et al., 2015; Conlon et al., 2018, 2019). By involving a transcriptomic approach, this study investigates the biochemical interplay between the JH and ecdysteroid pathways during the larval spinning stage while attempting to elucidate their role in *Varroa's* reproductive cycle. The differential fecundity of *Varroa* between worker and drone pupae in *A. mellifera*, combined with prior knowledge of *Varroa's* life history, can aid in the identification of genes linked to *Varroa's* reproductive success.

## 2 MATERIALS AND METHODS

### 2.1 Larvae sampling

The fieldwork took place in May and June 2018, at the apiary of the University of Agriculture Sciences and Veterinary Medicine, Cluj‐Napoca, Romania (46°45′N 23°34′E).

**FIGURE 1:**
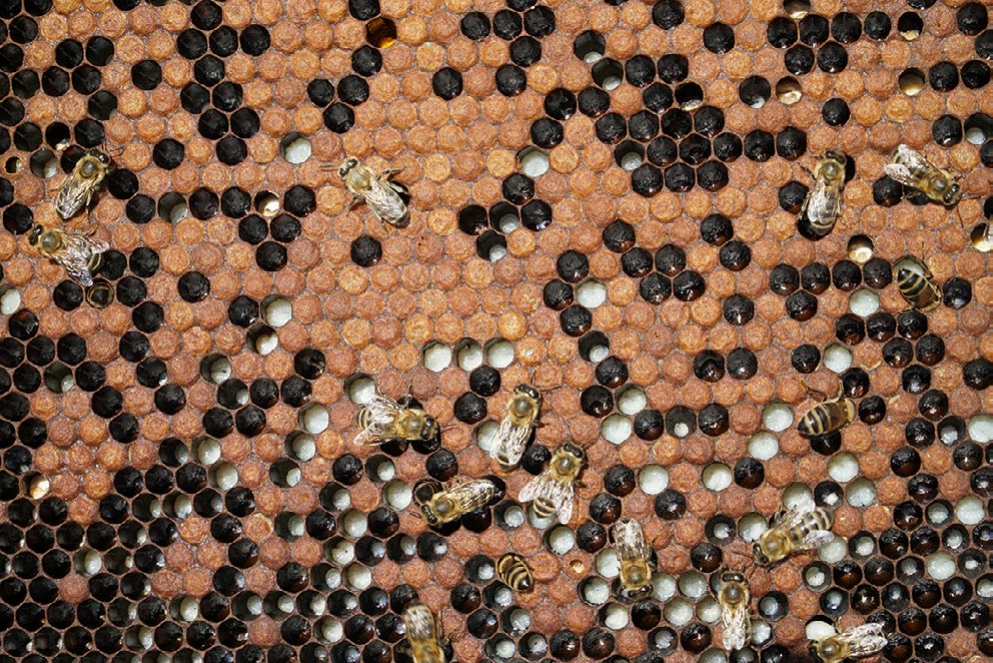
Brood comb containing worker progeny in different stages of development before and after capping together with nurse bees

Freshly capped larvae (Figure 1) from a colony of *A. mellifera* were traced using a transparent sheet (Human et al., 2013). When enough cells had been capped, the frame was placed in an incubator at 35°C and 70% humidity.

Fifth instar prespinning stage larvae from drones and workers were sampled right before capping (Time point (Tp) 0) and after cell capping at intervals 8, 12, 16, 24, and 30 hr (noted from now on Tp 8, Tp 12, Tp 16, Tp 24, and Tp 30, respectively). For each time point, except Tp 8 and Tp 30 where 10 were sampled, 5 larvae were carefully removed from their cell, snap‐frozen in liquid nitrogen, and stored at −80ºC for later RNA extraction. Each cell was inspected to ensure that it was not infested with *V. destructor*. We therefore only sampled uninfested pupal cells of *A. mellifera*.

### 2.2 RNA extraction

Total RNA was extracted from the entire body of the worker and drone larvae sampled at each Tp using QIAzol lysis reagent (Qiagen). The protocol supplied by the manufacturer was adjusted for the body weight of the drones and workers. The quantity and quality of RNA were assessed using a NanoDrop 2000 spectrophotometer (Thermo Fisher). Over 180 µg RNA free of contaminants were obtained per sample. A double checking was performed using the Agilent Bioanalyzer (Novogene), and the retrieved RIN values were in the following ranges: 9–9.8 for 27 samples, 8–8.9 for 33 samples, and 7.4–7.8 for 20 samples.

### 2.3 RNA‐seq and differential expression analysis

Library preparation and whole transcriptome sequencing was performed by Novogene (Hong Kong, China). The cDNA libraries were built with fragment sizes of 250–300 bp and sequenced using Illumina sequencing technology (NovaSeq, PE 2x150), producing an average of 24,586,613 sequences (sd = 2,927,419) per sample.

The raw RNA‐seq FASTQ paired‐end reads for each sample were aligned to the Amel_HAv3.1 *A. mellifera* reference genome assembly (GCA_00325439.2) (Wallberg et al., 2019).

The paired‐end sequence reads were estimated as fragment counts for each gene with the *featureCounts* program, part of the *Rsubread* package (Chen et al., 2016; Liao et al., 2014, 2019).

Differential gene expression (DGE) analysis between biological conditions (RNA extracted from drones and worker larvae at Tp 0 (brood cell precapping) and at Tp 8, Tp 12, Tp 16, Tp 24, and Tp 30 after brood cell capping) was based on the pipeline from Chen et al. (2016) and implemented in the R package *edgeR* (Robinson et al., 2010). The compositional biases between libraries were eliminated by a trimmed mean of M values (TMM) normalization in *edgeR*. The subsequent library read frequencies were normalized in the downstream analysis of differential expression between groups. The transcript abundance differences between samples were quantified by the log2‐fold change (logFC) values obtained in the paired contrasts between the experimental conditions. The genes associated with low read counts were excluded by setting the minimum count‐per‐million (CPM) values at 1 (cpm > 1.0) and in at least two libraries in each condition.

The logFC values for the genes related with molting and JH pathways (Tables 1 and 2) were extracted and further analyzed in this study. For assessing the true developmental stage of the larvae, the expression of fibroins was also analyzed. The fibroins are expressed during the larval spinning stage (Silva‐Zacarin et al., 2003) and are encoded by four genes in honey bees (Sutherland et al., 2006). Two types of comparisons between the biological conditions were performed; one of them shows the genes expression changing for each Tp relative to Tp0 in each sex, and the other one indicates the differences in drones relative to workers, at each time point. Only the significant changes between the different conditions were considered (adjusted *p* < 0.05).

### 2.4 RT‐qPCR

To validate the RNA‐seq results, a RT‐qPCR was performed for the *jhamt* gene. The reverse transcription was done for 2 µg RNA/sample, 5 samples/variant, using qPCRBIO cDNA synthesis kit (PCRBiosystems) and following the indications provided by the supplier. The qPCR was performed using the kit qPCRBIO SyGreen Mix Lo‐ROX (PCRBiosystems) on LightCycler 480 Real‐Time PCR System (Roche) set up for a denaturation step at 95°C for 2 min, followed by 40 PCR cycles of 95°C for 10 s, 56°C for 10 s, and 72°C for 20 s. Ribosomal protein L32 (*RpL32*, also known as *RP49*, NM_001011587.1) gene was used for normalization of the *jhamt* gene expression as its expression is stable during the development of honey bee larvae (Mackert et al., 2010). The primers for *RpL32*, F‐67 (5′‐TCCCATAACGTTCTATCTGTGGT‐3′) and R‐254 (5′‐AACGATCACTCTGGTGACGA‐3′) and *jhamt*, F‐393 (5′‐TTTGGACATAGGTTGCGGAC‐3′) and R‐578 (5′‐TTGGGCAAATCCATGGTCTC‐3′) were designed using Primer‐BLAST (NCBI). We analyzed the differences in relative gene expression between sexes and time by applying 2^−ΔΔCT^ method (Livak & Schmittgen, 2001) and using an ANOVA with Bonferroni post hoc test (STATISTICA 8.0).

### 2.5 Plotting and visualization

Plots were generated using “viridis” R package (https://github.com/sjmgarnier/viridis) and R package ggplot2 (Wickham, 2016).

## 3 RESULTS

To address the question of which are the available hormone‐related factors in postcapping honey bees development that could sustain *Varroa's* preferences for drones, a DGE analysis of the genes coding for the main enzymes involved in 20‐hydroxyecdysone (20E) and JH biosynthesis was performed in two ways. One is pointing out the changes in genes expression at different Tps during 30 hr postcapping (at 8, 12, 16, 24, and 30 hr), relative to the precapping Tp 0, for both drone and worker larvae. An analysis was also run comparing drones versus workers at each Tp. All the genes in the ecdysteroid and JH biosynthesis pathways considered for this study showed significant changes in DGE analysis with adjusted *p* < 0.05 (values provided in the supplementary files).

### 3.1 The ecdysteroid pathway

**FIGURE 2:**
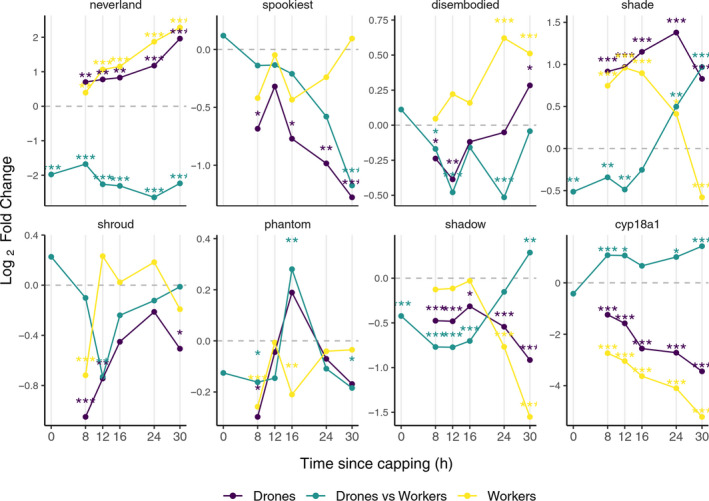
Differential gene expressions in molting hormone pathway. Differential expressions at Tp 8, 12, 16, 24, and 30 relative to Tp 0 for drone (indigo) and worker (yellow) larvae; drone versus worker differential expressions at Tp 0, 8, 12, 16, 24, and 30, respectively, are shown in green. Statistically significant logFC values for adjusted *p*: *p < 0.05, **p < 0.01, ***p < 0.001 (available in supporting information file, Sheets 1–3)

Among the Halloween genes expressed in the prothoracic gland, *neverland*, *shroud*, *spook*, *phantom*, *disembodied,* and *shadow* (Yamazaki et al., 2011), *neverland* was significant upregulated, relative to Tp 0 in both sexes at each Tp (Figure 2). Although both sexes showed a constant time‐associated upregulation in the expression of *neverland*, the transcript abundance proved to be higher in workers at every investigated Tp, including the reference Tp 0.

*Shadow* (LOC411893) was constantly downregulated to each Tp in drones, unlike in workers where a drop in gene expression occurred only at Tp 24 and Tp 30, respectively, as compared to Tp 0 (Figure 2). The difference between workers and drones is observed in the sex/time point associated comparative analysis (Figure 2): Tp 30 showed increased expression in drones relative to workers despite having been higher in workers in the earlier stages of development (upregulation in workers at Tp 0–Tp 16, respectively).

The remaining four intermediate genes, *shroud*, *spook*, *phantom,* and *disembodied*, showed either no significant upregulation or some Tp dependent fluctuations in expression, as would be the case of *phantom* in drones (Tp16) and *disembodied* in worker (Tp 24 and Tp 30) and drone larvae (Tp 30) (Figure 2).

The ecdysone release from the prothoracic gland is activated to 20‐hydroxyecdysone (20E) in the peripheral tissues by the *shade* gene (Yamazaki et al., 2011). This was significantly upregulated after cell capping in both sexes and remains like that in drones for all the Tps. In worker larvae, its level starts to decrease after Tp 24 (Figure 2). In the drone versus worker analysis, *shade* showed a similar pattern of upregulation to *shadow,* toward the ending of the time interval studied (Tp24 and Tp30), being more active in drone larvae.

The *cyp18a1* gene, known to be engaged in 20E degradation (Rewitz et al., 2010), was downregulated in both sexes starting at Tp 8 (Figure 2). However, comparative analysis between sexes indicates a consistently higher level of expression in drones relative to workers.

### 3.2 JH pathway genes

**FIGURE 3:**
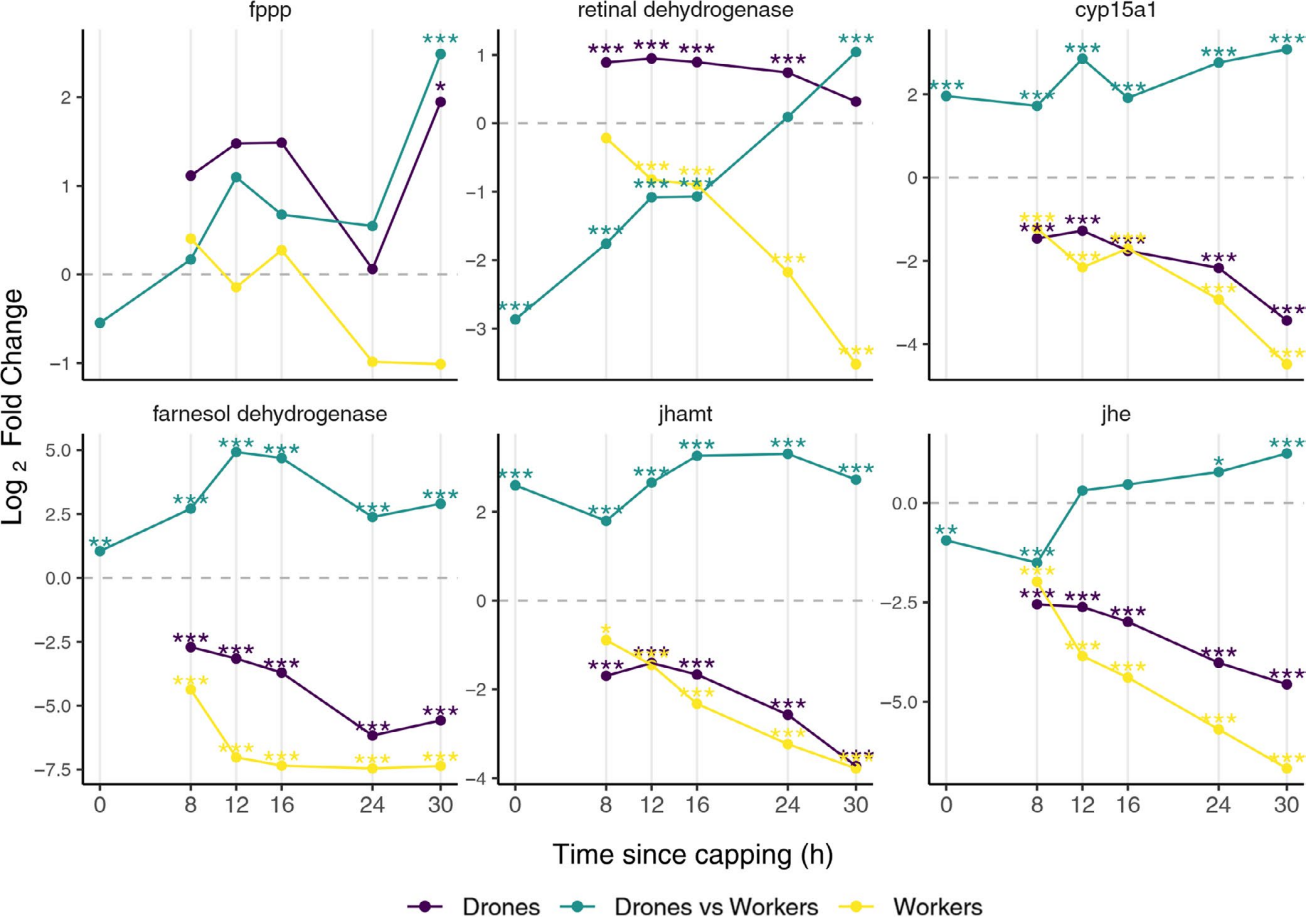
Differential gene expression in Juvenile hormone pathway. Differential expressions at Tp 8, 12, 16, 24, and 30 relative to Tp 0 for drone (indigo) and worker (yellow) larvae; drone versus worker differential expressions at Tp 0, 8, 12, 16, 24, and 30, respectively, are shown in green. Statistically significant logFC values for adjusted *p*: *p < 0.05, **p < 0.01, ***p < 0.001 (available in supporting information file, Sheets 4–6)

The JH pathway associated genes were generally downregulated for each Tp relative to Tp 0 in both drone and worker larvae. The exceptions to this were *fppp* at Tp 30 and *retinal dehydrogenase* genes in drones (Figure 3). The *retinal dehydrogenase* was upregulated in drones from Tp 8 to Tp 24, followed by a drop in expression at Tp 30 to a level which was not significantly different from that observed at Tp0.

**FIGURE 4:**
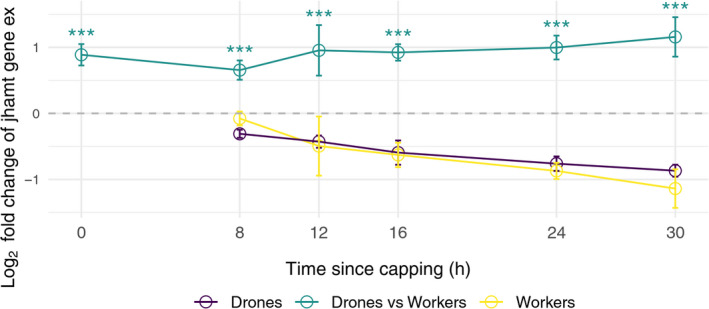
Relative gene expression for *jhamt* gene. Expression of *jhamt* at Tp 8, 12, 16, 24, and 30 relative to Tp 0 for drone (indigo) and worker (yellow) larvae; drone versus worker relative expression at Tp 0, 8, 12, 16, 24, and 30, respectively, is shown in green, ***p < 0.001 (supporting information file, Sheet 10)

The overall downregulation of each gene in larvae at every time point after Tp 0 supports the preparation for ending of the antagonistic activity of JH during ecdysteroid pathway activation for assuring the transition to prepupal stage. However, the male–female comparative analysis revealed consistently higher expression levels of drone genes as compared to workers in three of the six genes (*farnesol dehydrogenase*, *jhamt*, *cyp15a1*); however, all genes were significantly upregulated in drones after 30 hr (Figure 3). The results obtained in transcriptomic analysis for *jhamt* gene were confirmed by RT‐qPCR which also shows a significant upregulation (*p* < 0.001) in drones relative to workers for all the time points (Figure 4 and Supplementary file). The gene *jhe*, which specifically degrades JH (Bomtorin et al., 2014; Mackert et al., 2008), was downregulated in both drones and workers, albeit to a lesser extent in drones (Figure 3). The significant upregulation of JH genes in drones suggests the existence of higher level of JH which could count for the prolongation of larval stage in drones.

### 3.3 Fibroins

**FIGURE 5:**
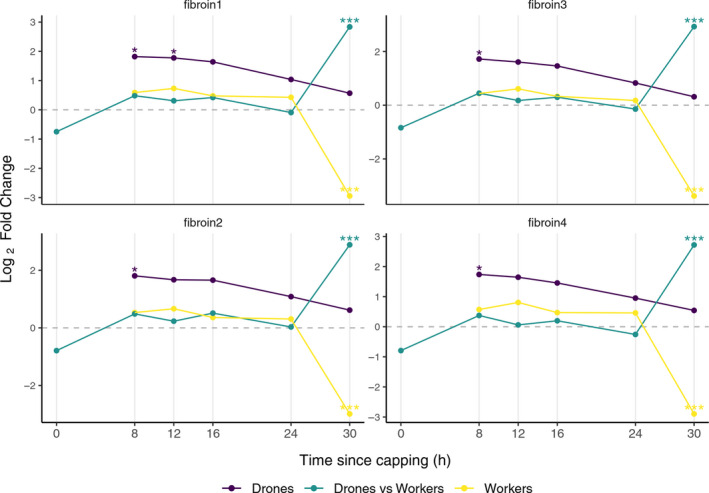
Differential gene expression of fibroins. Differential expressions at Tp 8, 12, 16, 24, and 30 relative to Tp 0 for drone (indigo) and worker (yellow) larvae; drone versus worker differential expressions at Tp 0, 8, 12, 16, 24, and 30, respectively, are shown in green. Statistically significant logFC values for adjusted *p*: *p < 0.05, ***p < 0.001 (available in supporting information file, Sheets 4–6)

Silk production in honey bees correlates well with the spinning stage of the larvae (Silva‐Zacarin et al., 2003; Sutherland et al., 2006), and the level of transcription of fibroins can be used as an indicator for this larval stage. Compared to Tp 0, the expression of all fibroins was slightly increased in drone larvae, while in workers the level of expression remains unchanged up to Tp 24, suffering a sudden drop at Tp 30 (Figure 5). The drones versus workers comparison shows a synchronous prolongation of sustained fibroins transcription at up to Tp 30, in drones (Figure 5). These results indicate a delay in drones for entering in the prepupal stage comparing to the worker larvae.

## 4 DISCUSSION

Our study systematically analyzed the expression of molting and JH‐associated genes in honey bees worker and drone larvae in the first 30 hr following the cells capping with a view to identifying compounds, which may be linked to the successful reproduction of *Varroa*. From this perspective, our analysis of JH cascade revealed perhaps the most interesting link.

**FIGURE 6:**
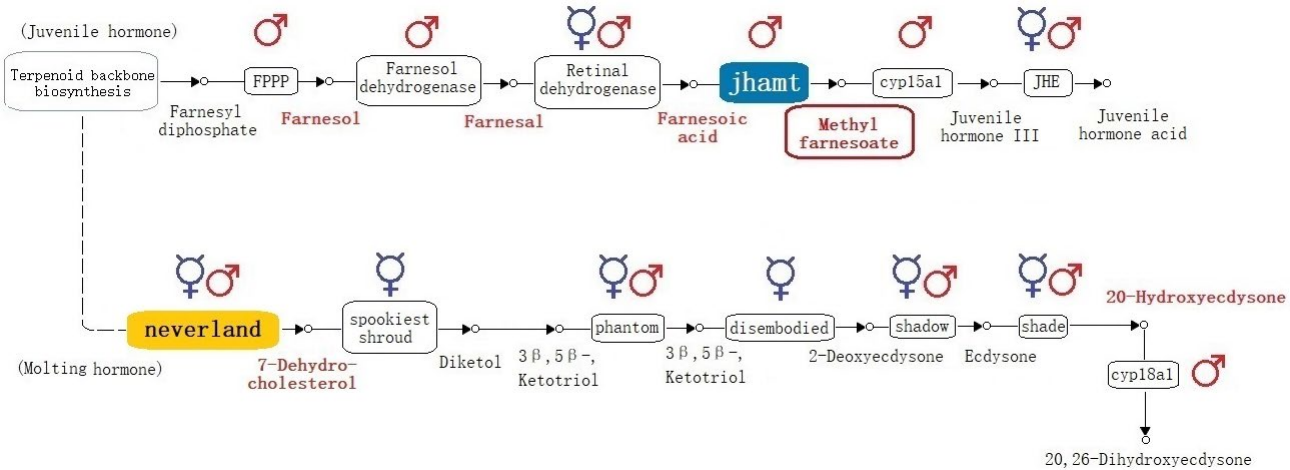
The prevalence of molting and juvenile hormone metabolites in honey bee larvae in the first 30 hr postcapping. The male (♂) and female (☿) symbols are indicating the genes for the enzymes that are upregulated in drones and worker larvae, in time and/or sex‐related analysis. The key enzymes in molting and juvenile hormone pathway that could have implication in Varroa's reproduction are highlighted in yellow and blue, respectively; the metabolites that are more prevalent or not restrictive (*neverland*) in drones are written in red. The hormone pathways are modified from KEEG (Kanehisa et al., 2016)

The majority of genes in the JH pathway showed a consistent downregulation in both sexes over time (Figure 3). This generally decreased expression during the whole time frame supports the antagonistic action of JH to ecdysteroids pathway (Liu et al., 2018) as the larvae enters the prepupal stage (Riddiford, 2012). By comparing drone and worker larvae, we identified significant upregulation of JH genes in drones for the majority of the Tps (Figure 3, Figure 6), indicating that the JH metabolites are at higher level in drones. Biochemical analysis of the JH in honey bee's development has shown that it decreases right before capping (Rachinsky et al., 1990). Although fluctuating, during the first 30 hr after cell sealing, the level of JH is higher in drones when compared to workers (Hanel & Koeniger, 1986; Rosenkranz et al., 1993). Our findings support these trends.

The main products of JH pathway are the two key hormones methyl farnesoate (MF), synthesized by JH acid O‐methyltransferase (*jhamt*), and JH, synthesized via MF epoxidase (*cyp15a1*) (Li et al., 2013) (Figure 6). MF is the immediate precursor of JH (Nagaki et al., 2009) and has been detected in at least five orders of insects (Teal et al., 2014). Special emphasis should therefore be placed on *jhamt* gene activity, which is crucial to the production of these two hormones. From Tp 0, *jhamt* shows a sixfold increase in drones as compared to workers and remains at higher levels, in drones, throughout the entire test period (with 6.04‐; 3.46‐; 6.3‐; 9.57‐; 9.86‐; and 6.6‐fold increased absolute values at Tp 0; 8; 12; 16; 24; and 30, Figure 3). The higher male‐associated expression in *jhamt*, throughout the first 30 hr postcapping, raises the possibility it may influence *Varroa's* reproduction. Considering the potential ecological implications, MF has physical, chemical, and functional properties that would make it a potential kairomone. Unlike ecdysteroids, MF is a volatile compound that can act as pheromone (Břízová et al., 2015; Ho & Millar, 2001; Tanaka et al., 1994) as well as a hormone and precursor of JH (Wen et al., 2015). Higher expression of *jhamt* in precapped drones (Tp 0) therefore provides the opportunity for it to function as a kairomone, attracting *Varroa* into the drone brood cells. Furthermore, the consistently higher levels of *jhamt* in drones during the first 30 hr postcapping could indicate a potential role for MF in ovogenesis, with elevated levels for a longer period of time in drones increasing *Varroa's* fecundity. Although *cyp15a1* exhibits a similar trend in gene expression (Figure 3), suggesting the presence of an elevated turnover rate of MF into JH, the conflicting results reported in previous studies (Rosenkranz et al., 1990, 1993) do not fully support the active involvement of this hormone in the initiation of *Varroa's* reproductive cycle as it was not possible to detect JH in *Varroa* (Cabrera et al., 2009; Cabrera et al., 2009; Qu et al., 2015).

In crustaceans, vitellogenesis has been shown to be induced by MF (reviewed by Cabrera, Donohue, & Roe, 2009) and, indeed, MF is known as the key JH‐like hormone in chelicerata and crustaceans (reviewed by Qu et al., 2015). Thus, MF could represent an alternative to JH for the initiation of ovogenesis in *Varroa*. MF epoxidase, also higher in drones, acts exclusively on MF, and its expression is upregulated when JH is produced (Helvig et al., 2004). It has been observed that pesticide treatments reduce the MF levels in freshly emerged honey bee workers without affecting the levels of JH (Schmehl et al., 2014), suggesting the existence of excessive MF titers in honey bee pupae. This is indeed the case in *Drosophila melanogaster* hemolymph, where the MF concentration was found to reach 50 times higher levels than JH in newly emerged individuals (Teal et al., 2014). It is therefore unsurprising that comparative analyses of JH level, which is very low but similar in worker larvae of European honey bees, *A. mellifera lamarckii*, Africanized honey bees and the Asian honey bee (*A. cerana*) (Rosenkranz et al., 1990, 1993), could not explain why *Varroa* reproduces successfully in the worker pupae of European honey bees, has reduced fecundity in *A. mellifera lamarckii* and Africanized honey bees, and does not reproduce at all in the worker pupae of *A. cerana* (Rosenkranz et al., 1990, 1993); it may not have been the correct JH‐like hormone to study. While there are no available data to show that MF titers mirror the level of JH in honey bee larvae, it could be possible that a minimal threshold of MF is needed for *Varroa* to activate its ovaries. Considering the volatility of MF, we can say that the size of the larvae, and the length of time for which MF is upregulated in drone pupae, could make a difference in fulfilling that threshold level. *A. cerana*, *A. mellifera lamarckii,* and Africanized honey bees are all significantly smaller than the European honey bees (Dyer & Seeley, 1987; Michelette & Engels, 1995; Schmolz et al., 2001), while *A. cerana* workers also have a shorter pupation time (Koetz, 2013). This may have an impact on the MF titer in the brood cell and constrain or elevate *Varroa's* fecundity. Support for this can be found when considering the life history of *Varroa's* reproductive cycle. *Varroa* invades the cells toward the end of the feeding period of the honey bee larvae when they are about to reach the highest size and become more easily to be sensed by *Varroa* (Boot et al., 1995). *Varroa* is then only able to complete its reproductive cycle as long as the cell remains closed (Oddie et al., 2018). *Varroa's* fecundity is therefore higher in the larger and longer‐pupating drones which have a higher JH (and subsequently more MF) titer than the worker larvae (Hanel & Koeniger, 1986; Rosenkranz et al., 1993).

Upstream of *jhamt*, *farnesol dehydrogenase* was also a significantly upregulated gene in drones relative to workers at all tested time points (Figure 3). This enzyme is involved in the synthesis of farnesal, another volatile compound, which can act as pheromone in insects (Zagatti et al., 1987) and is a precursor of MF. The significance of *farnesol dehydrogenase* for *Varroa* was previously investigated as it is upregulated in the worker larvae infested with *Varroa* and infected with DWV (Erban et al., 2019). *Fppp* and *retinal dehydrogenase*, both upregulated in drone larvae relative to workers toward the end of the tested period, are responsible for production of farnesol and farnesoic acid, respectively. Both substances are known pheremones (Breeden et al., 1996; Schmitt et al., 2007). Considering our results, we argue that the JH biosynthesis pathway offers suitable kairomone candidates that could influence *Varroa's* reproduction, particularly in relation to elevated fecundity in drone pupal cells.

During our temporal distribution approach, the activity of the ecdysteroid biosynthesis pathway showed a switch on (Figure 2), indicating a sustained hormone requirement during the 30 hr postcapping interval, in both sexes. Comparative analysis between sexes revealed a delay in gene upregulation in drones (*shadow* and *shade*, Figure 2). The upregulation of genes involved in the ecdysteroid synthesis in both sexes was confirmed by the constant decrease in *cyp18a1* gene expression, known to encode the enzyme responsible for degrading 20E (Rewitz et al., 2010). The lesser extent of decreasing of 20E degrading enzyme in drones may indicate that the titer of active form ecdysone is higher in drone larvae (Conlon et al., 2019; Rewitz et al., 2010), something which was also highlighted by Hartfelder and Engels (1998). In Acari, ecdysteroids are involved in regulation of vitellogenesis (Cabrera et al., 2015) and ecdysteroid‐related genes associated with *V. destructor*‐resistant honey bees have been identified in populations from Gotland, Sweden (Conlon et al., 2018) and Toulouse, France (Conlon et al., 2019). Among the Halloween genes, the homologs of *neverland* and *Cyp18a1* (Techer et al., 2019) have not been identified in the *Varroa* genome while *Cyp18a1* gene is believed to have been lost in the Acari (Grbić et al., 2011). It is therefore highly unlikely that *Varroa* is able to synthesize the ecdysteroids ecdysone and 20E but both of these hormones have been identified from *Varroa* (Feldlaufer & Hartfelder, 1997) and analogues of ecdysone have been shown to stimulate vitellogenesis in *Varroa* (Cabrera et al., 2017). There is therefore strong evidence that *Varroa* requires and uses host‐produced metabolites of ecdysteroids pathway to initiate the reproductive cycle. The upregulation of *neverland* in both honey bees sexes immediately after capping imply the availability of its product is not restrictive for *Varroa* in either sex in *A. mellifera*.

The slower rate at which the JH pathway is downregulated in drones could be linked to their longer pupation time (Hrassnigg & Crailsheim, 2005; Jay, 1963). The timing of metamorphosis is controlled by JH level, and treatment with JH analogue methoprene has been found to delay the larval to pupal transition (Minakuchi et al., 2008). In *Tribolium castaneum*, pupation was found to be induced via *jhamt* mandatory gene suppression (Minakuchi et al., 2008). To further verify whether the results of hormone‐related gene expression are reflected by the true developmental stage of the larvae, we analyzed the expression of fibroins, the main components of the honey bees silk (Hepburn et al., 2013). Silk is produced from middle of the 5th instar larval period and is deposited first in the salivary glands before being distributed during the spinning process after the cell has been closed (Silva‐Zacarin et al., 2003). Fibroin biosynthesis is regulated by a cross‐talk between JH and ecdysone (Liu et al., 2019) with JH increasing the transcription in the condition of a relatively low ecdysone background. In the present study, the higher activation of fibroins transcription starting with Tp 8 in drones and prolongation of synthesis up to Tp 30, so later than in workers, confirms the projuvenile status reflected by the extension of the spinning larval stage in drones exactly as it was suggested by the higher level of JH‐related genes expression. The longer postcapping windows—up to 36 hr in drones versus 12 hr in worker larvae—in which mite reproduction suffers no inhibition (Frey et al., 2013) could also be explained by this delay in JH‐related downregulation in drones.

We propose that an increased *jhamt* gene expression in honey bee drones could be linked to a hormonal threshold required by the female mite to start ovogenesis and her reproductive cycle. The analysis of the JH pathway from another perspective may inspire future studies for finding additional molecules that are involved in the complex interplay between host and parasite in this system and may shed new light on ways in which *Varroa* infestation can be combated.

## CONFLICT OF INTEREST

None declared.

## AUTHOR CONTRIBUTIONs

**Cristian M. Aurori:** Conceptualization (equal); data curation (lead); formal analysis (lead); methodology (equal); software (lead); visualization (equal); writing – original draft (equal); writing – review and editing (supporting). **Alexandru‐Ioan Giurgiu:** Investigation (supporting); methodology (supporting); visualization (supporting); writing – original draft (supporting); writing – review and editing (supporting). **Benjamin H. Conlon:** Conceptualization (supporting); methodology (supporting); writing – original draft (supporting); writing – review and editing (equal). **Chedly Kastally:** Data curation (supporting); visualization (equal); writing – original draft (supporting); writing – review and editing (equal). **Daniel S. Dezmirean:** Funding acquisition (supporting); methodology (supporting); project administration (equal); supervision (equal); writing – original draft (supporting); writing – review and editing (supporting). **Jarkko Routtu:** Conceptualization (equal); funding acquisition (supporting); methodology (supporting); writing – original draft (supporting); writing – review and editing (equal). **Adriana Aurori:** Conceptualization (equal); investigation (lead); methodology (equal); project administration (equal); supervision (equal); visualization (equal); writing – original draft (equal); writing – review and editing (equal).

## Supporting information

Supplementary Material

Supplementary Material

## Data Availability

The data that supports the findings of this study are available at https://doi.org/10.5061/dryad.prr4xgxk9 with additional material to follow after a 1 year embargo, due to third party constraints.
