# Supplementary material for: Juvenile hormone pathway in honey bee larvae: A source of possible signal molecules for the reproductive behavior of Varroa destructor
Source: Ecol Evol. 2020 Dec 21;11(2):1057–68. doi: 10.1002/ece3.7125 (PMC7820148; doi:10.1002/ece3.7125)
Supplement: Supplementary file 2 — Supplementary Material [file ECE3-11-1057-s002.docx]

**Supporting information:**

**All the supporting information is available into one excel file in the following 10 sheets:**

**Sheets 1-3: Molting hormone pathway: differential gene expressions between time points in Drones (Sheet 1), Workers (Sheet 2);** **differential gene expressions between Drones and Workers at each time point (Sheeet 3);** Gene IDs, log2-fold changes (logFC) and statistical significance (P values).

**Sheet 4-6: Juvenile hormone pathway: differential gene expressions between time points in Drones (Sheet 4) and Workers (Sheet 5);** differential gene expressions between Drones and Workers at each time point (Sheet 6); Gene IDs, log2-fold changes (logFC) and statistical signifficance (P values).

**Sheet 7-9**: Fibroins: differential gene expressions between time points in Drones (Sheet 7) and Workers (Sheet 8); differential gene expressions between Drones and Workers at each time point (Sheet 9); Gene IDs, log2-fold changes (logFC) and statistical significance (P values).

**Sheet 10**: The results of statistical analysis for *jhamt* relative expression during temporal and drone versus worker analysis. The significant p values for ANOVA and Bonferroni post hoc are highlighted in yellow.
